# Precision Measurement of Fundamental Constants Using GAMS4

**DOI:** 10.6028/jres.105.003

**Published:** 2000-02-01

**Authors:** M. S. Dewey, E. G. Kessler

**Affiliations:** National Institute of Standards and Technology, Gaithersburg, MD 20899-0001, USA

**Keywords:** atomic masses, binding energy, crystal diffraction, fundamental constants, gamma rays, molar Planck constant, neutron mass

## Abstract

We discuss the connection of high-energy gamma-ray measurements with precision atomic mass determinations. These rather different technologies, properly combined, are shown to lead to new values for the neutron mass and the molar Planck constant. We then proceed to describe the gamma-ray measurement process using the GAMS4 facility at the Institut Laue-Langevin and its application to a recent measurement of the 2.2 MeV deuteron binding energy and the neutron mass. Our paper concludes by describing the first crystal diffraction measurement of the 8.6 MeV ^36^Cl binding energy.

## 1. Introduction

The binding energy of the last neutron in a nucleus can be assessed by determining the sum of gamma-ray transition energies along a path from the capture state to the nuclear ground state. This binding energy is associated with a corresponding mass decrement by the Einstein relation, *E* = *mc*^2^. We begin by exploring the structure of this connection and its consequences (Sec. 2). Thereafter we describe the apparatus and procedures for accurate gamma-ray wavelength measurements by the GAMS4 facility at the Institut Laue-Langevin (ILL) (Sec. 3). In the remainder of the paper (Sec. 4), we summarize recent applications of GAMS4 to a measurement of the 2.2 MeV binding energy of deuterium and the neutron mass and to the first crystal diffraction measurement of the 8.6 MeV ^36^Cl binding energy.

## 2. The Connection Between Gamma-Ray Energies and the Fundamental Constants

In recent years, a new mass spectroscopic technique that measures atomic masses by comparing the cyclotron frequencies of two different trapped ions has been developed. The results obtained are more than an order of magnitude more accurate than previous atomic mass determinations [1, 2]. This dramatic reduction in the uncertainty of atomic mass measurements has renewed interest in the connection between atomic masses and precisely measured wavelength intervals that can lead to precise values for the neutron mass and the molar Planck constant. In Sec. 2.1 we describe this connection in the context of present day uncertainties. In Sec. 2.2 we describe a speculative framework within which the relationship between atomic mass and gamma ray measurements has new significance.

### 2.1 GAMS4 and the Molar Planck Constant

Two definitions are critical for the arguments that follow: the atomic mass unit and the Avogadro constant. One atomic mass unit (u) is defined as 1/12 times the mass of a ^12^C atom. The related Avogadro constant *N*_A_ is defined as the number of atoms present in 12 g of ^12^C (thus one mole of ^12^C atoms weighs exactly 12 g).

We begin by considering a typical neutron capture reaction

$$n{+}^{A}X\to^{A+1}X+\gamma ’s.$$ (1)

Energy conservation applied to this reaction yields

$$[A_{r}(n)u]c^{2}+[A_{r}{(}^{A}X)u]c^{2}=[A_{r}{(}^{A}X)u]c^{2}+\frac{hc}{\lambda_{A+1}^{*}}$$ (2)

where *A*_r_ is the unitless relative atomic mass (*A*_r_(^12^C)=12), u is the mass of an atomic mass unit, h is the Planck constant, c is the speed of light, and $\frac{1}{\lambda_{A+1}^{*}}$ represents the sum of reciprocal wavelengths over those gamma-rays which connect the *^A^*^+1^X capture state to the ground state (typically 3 gamma-rays for GAMS4 measurements). Several things are worth noting in this equation:

- The experiments described in Refs. [1] and [2] measure *A*_r_
- $u={\left\{\frac{10^{-3}}{N_{A}}\right\}}_{\mathrm{SI}}$ where {}_SI_ indicates the numerical value of the quantities contained in the curly brackets when expressed in their respective SI units
- In a neutron reactor the characteristic energy of the neutron is 0.06 eV (the target atom is at rest); this kinetic energy introduces a small neutron incident angle dependent correction to our analysis; if, as happens experimentally, there is averaging over the angle, a tiny second order correction remains (1.3×10^−8^ for neutron capture on hydrogen)
- $\lambda_{A+1}^{*}$ includes a correction for the *^A^*^+1^X recoil energy

Rearranging Eq. (2) we obtain

$$A_{r}(n)+A_{r}{(}^{A}X)-A_{r}{(}^{A+1}X)={\left\{10^{-3}\frac{N_{A}h}{c}\right\}}_{\mathrm{SI}}{\left\{\frac{1}{\lambda_{A+1}^{*}}\right\}}_{\mathrm{SI}}$$ (3)

This equation expresses a relationship between mass measurements in atomic mass units and wavelength measurements in meters. We adopt the viewpoint that it contains three types of “measurable” quantities: *A*_r_, *λ*, and *N*_A_*h*/*c*. We consider the experimental uncertainties on each of these three quantities in turn. All uncertainties given in this paper are one standard deviation estimates.

The conversion factor that connects atomic mass measurements with wavelength intervals in Eq. (3) is the molar Planck constant *N*_A_*h* divided by *c*, where *c* is an exactly defined quantity. Although a value for this conversion factor with a relative uncertainty of 9.0×10^−8^ is available from Ref. [3], a significantly more accurate value can de derived from recent measurements of fundamental constants. It can be shown that [4]

$${\left\{10^{-3}\frac{N_{A}h}{c}\right\}}_{\mathrm{SI}}={\left\{\frac{A_{r}(e)\alpha^{2}}{2R_{\infty }}\right\}}_{\mathrm{SI}}$$ (4)

where

*A*_r_(e): = relative atomic mass of the electron
  = 5.485 799 111(12)×10^−4^ [5]
*α*^−1^: = inverse fine-structure constant
  = 137.035 999 58(52) [6]
*R*_∞_: = Rydberg constant
  = 10 973 731.568 639(91) m^−1^ [7]

which leads to the conversion factor

$$1\;m^{-1}=1.331\;025\;045(11)\times 10^{-15}u$$ (5)

(relative uncertainty of 7.9×10^−9^). The dominant source of error in this expression comes from the fine structure constant [6].

As discussed in Sec. 4, the GAMS4 facility (described in Sec. 3) is now capable of measuring $\lambda_{A+1}^{*}$ in a few nuclei with a relative estimated standard deviation *u*_r_ ≈ 2×10^−7^ while the mass doublet ratios measurements described in Refs. [1] and [2] may soon reach an uncertainty *u*_r_ ≈5×10^−11^. For typical values of the atomic weight, A, this translates into an uncertainty *u*_r_ ≈ 2×10^−7^ on the quantity *A*_r_(n) + *A*_r_(*^A^*X) − *A*_r_*u*_r_(*^A^*^+1^X).

Evidently, *N*_A_*h/c* can be considered as either: 1. An otherwise established numerical value; or 2. An object to be measured. The consequences are then as follows:

#### When N_A_h/c is taken as given

Given the small uncertainty on *N*_A_*h/c* it is reasonable to proceed from the viewpoint that its value is certain. In that case, Eq. (3) can be used to calibrate wavelength intervals using mass measurements as input, or to check atomic mass intervals using wavelength measurements as input (the relative uncertainties in both measured quantities are comparable). However, one other issue must be addressed. Equation (3) contains the neutron mass that cannot be measured with a trap. However, it can be determined, and subsequently eliminated from consideration, if the special case of neutron capture on hydrogen is considered

$$A_{r}(n)+A_{r}(H)-A_{r}(D)={\left\{10^{-3}\frac{N_{A}h}{c}\right\}}_{\mathrm{SI}}{\left\{\frac{1}{\lambda_{D}^{*}}\right\}}_{\mathrm{SI}}$$ (6)

where D refers to deuterium or ^2^H. Our recent measurement of the deuteron binding energy S(D) (or equivalently $\lambda_{D}^{*}$) combined with Eq. (5) and mass spectroscopic measurements of *A*_r_(H) and *A*_r_(D) yields a precise value for *A*_r_(n) (see Sec. 4.1).

#### When N_A_h/c is to be measured

Despite the small uncertainty on *N*_A_*h/c* we may view Eq. (3) as providing a means to measure it. To extract the molar Planck constant, we start by subtracting Eq. (3) from Eq. (6) to obtain

$$A_{r}{(}^{A+1}X)+A_{r}(H)-A_{r}{(}^{A}X)-A_{r}(D)={\left\{10^{-3}\frac{N_{A}h}{c}\right\}}_{\mathrm{SI}}{\left\{\frac{1}{\lambda_{D}^{*}}-\frac{1}{\lambda_{A+1}^{*}}\right\}}_{\mathrm{SI}}$$ (7)

where {}_SI_ is implied for all wavelengths. Practical considerations of this conceptual equation lead to an optimum strategy for obtaining ${\left\{10^{-3}\frac{N_{A}h}{c}\right\}}_{\mathrm{SI}}$.

The atomic mass difference on the left side is obtained from ion mass ratios by taking into account chemical binding energies and ionization energies. To illustrate how the uncertainty of the ion mass ratio translates into an uncertainty of the atomic mass difference, we use the mass doublet ratio *^A^*XD^+^/*^A^*^+1^XH^+^ as an example [8].

$$r=\frac{A_{r}{(}^{A}{\mathrm{XD}}^{+})}{A_{r}{(}^{A+1}{\mathrm{XH}}^{+})}=\frac{A_{r}{(}^{A}X)+A_{r}(D)-A_{r}(e)+A_{r}(\Delta E_{1})}{A_{r}{(}^{A+1}X)+A_{r}(H)-A_{r}(e)+A_{r}(\Delta E_{2})}$$ (8)

where Δ*E*_1_ and Δ*E*_2_ are the appropriate chemical binding and ionization energies. This equation is easily translated into the mass difference

$$A_{r}{(}^{A+1}X)+A_{r}(H)-A_{r}{(}^{A}X)-A_{r}(D)=(1-r)[A_{r}{(}^{A+1}X)+A_{r}(H)-A_{r}(e)]+A_{r}(\Delta E_{1})-rA_{r}(\Delta E_{2})$$ (9)

Although *A*_r_(*^A^*^+1^X) and *A*_r_(H) appear on the right hand side of this equation, they are multiplied by (1−*r*) which is typically on the order of 10^−3^ and thus do not need to be known very precisely. The uncertainty of the mass difference is essentially the uncertainty of the small factor (1−*r*). Since the mass difference on the left hand side is typically in the 6 to 9×10^−3^ range, the factor (1−*r*) becomes smaller as the mass *A* increases. Thus, for a given relative uncertainty of the ion mass doublet ratio, *r*, the relative uncertainty of (1−*r*) and the atomic mass difference increases with increasing *A*.

The wavelengths on the right hand side of Eq. (7) are obtained from GAMS4 measurements. The magnitude of $1/\lambda_{A+1}^{*}$ is typically 3 to 5 times larger than that of $1/\lambda_{D}^{*}$ and the relative uncertainty of the $\lambda_{A+1}^{*}$ and $\lambda_{D}^{*}$ are nearly the same. Thus the relative uncertainty of the right hand side is dominated by the relative uncertainty of $\lambda_{A+1}^{*}$. The relative uncertainty of the GAMS4 measurements is small when the binding energy interval *S*(*^A^*^+1^X)−*S*(D) is large.

The molar Planck constant in combination with other quantities whose values are very well known leads to a determination of the fine structure constant, *α*, as is shown by the following equation

$$\alpha^{-1}={\left[\frac{M_{e}}{2R_{\infty }\left(\frac{N_{A}h}{c}\right)}\right]}^{1/2}$$ (10)

where *M*_e_ is the electron molar mass and *R*_∞_ is the Rydberg constant. Note the similarity of Eqs. (4) and (10). The relative uncertainty of *α* from Eq. (10) is dominated by the molar Planck constant (*u*_r_ ≈ 1 to 2×10^−7^) and is to a good approximation one half the relative uncertainty of the molar Planck constant. However, there are a number of other ways to determine *α* which have already achieved a relative uncertainty less than 1×10^−7^. Table 1 shows the other major experimental approaches to the determination of *α*. The physics contained in these equations is extremely varied. Consistency among the different approaches probes both the reliability of the experiments and the existence of new physics.

Table 2 gives the magnitude of the relative uncertainty contributions from the mass difference and the GAMS4 wavelength measurements to the molar Planck constant for several nuclei. In addition, the total relative uncertainty contributions to the molar Planck constant and *α* are listed. Because of the low efficiency of GAMS4, the list of candidate nuclei is rather short. In every case except nitrogen, the two uncertainty contributions are comparable. In the case of nitrogen the mass difference contribution is quite small. The binding energy interval is also quite large (10.8−2.2 = 8.6 MeV). For these reasons, a precise measurement of *S*(^15^N) is highly desirable. Unfortunately the intensities of the gamma rays in nitrogen are quite low and it remains to be seen if a measurement in that system is possible. At present, it appears that Cl, S, and Si are promising candidates.

### 2.2 A Test of Special Relativity: Does *E = mc* ^2^?

In this section we describe a unique feature of the connection between atomic mass measurements and gamma-ray wavelength measurements in the context of a test of special relativity. A more detailed discussion is found in Ref. [14]. According to the special theory of relativity

- The rest energy *E* of a particle of mass *m* is given by $E=mc_{m}^{2}$, where *c*_m_ is the limiting velocity of a massive particle
- the energy of a photon of frequency *v* is given by $E=hv=\frac{hc_{\mathrm{em}}}{\lambda }$ where *c*_em_ is the velocity of propagation of an electromagnetic wave in vacuum.
- *c*_m_ = *c*_em_ = *c*

Michelson-Morley and Hughes-Drever experiments look for anomalous velocity-dependent effects that arise from motion of a test system with respect to a preferred frame. Tests of the equality of *c*_m_ and *c*_em_ are another class of tests that are sensitive to a violation of special relativity and a preferred frame. The basic test will be

$$\Delta mc_{m}^{2}=\frac{hc_{\mathrm{em}}}{\lambda }$$ (16)

in which a photon of wavelength *λ* is emitted in a transition where a mass Δ*m* is converted into electromagnetic radiation.

The test is most conveniently formulated in terms of two fine structure constants

$$\alpha_{m}=\frac{e^{2}}{\hbar c_{m}}\text{and}\;\alpha_{\mathrm{em}}=\frac{e^{2}}{\hbar c_{\mathrm{em}}}$$ (17)

where it is assumed that *e* and *ħ* are universal constants. A careful analysis reveals that the *α* obtained via the von Klitzing constant is *α*_em_ (Eq. (12) in Table 1) while *α* derived via GAMS4 and the mass difference measurements is a realization of *α*_m_. This could ultimately provide a determination of $\left(1-\frac{c_{m}}{c_{\mathrm{em}}}\right)$ with an error at the level of (1 to 2)×10^−7^. Currently the most accurate realization of *α*_m_ comes from the Rydberg constant *R*_∞_ and the Compton wavelength of the electron *λ*_C_ through the relation

$$\alpha_{m}=\sqrt{2R_{\infty }\lambda_{C}}$$ (18)

It yields

$$\left(1-\frac{c_{m}}{c_{\mathrm{em}}}\right)=1(12)\times 10^{-6}$$ (19)

Unlike other tests of special relativity, this limit does not depend on assumptions concerning the motion of the laboratory with respect to a preferred frame.

## 3. The GAMS4 Facility

The GAMS4 gamma-ray spectroscopy facility is a two axis flat crystal spectrometer located at the high flux reactor of the ILL. The instrument is used to study radiation produced in neutron capture reactions and is coupled to a reactor port which is specially equipped to transport and hold sources in a position tangential to the reactor core. Precision gamma-ray measurements which impact the fundamental constants require knowledge of two quantities, the lattice spacing, *d*, and the diffraction angle, *θ*. These two quantities are combined using the Bragg condition for diffraction, *λ* = 2*d*sin(*θ*), to obtain the wavelength, *λ*, of the gamma ray. In a typical neutron capture reaction *n* + *^A^*X → *^A^*^+1^X + *γ*, the wavelength or energy of the gamma ray is related to the mass of the neutron and the atomic masses of the source and product species. This link between the gamma-ray wavelengths and the atomic masses leads to certain fundamental constants as described above. The Bragg angles through which the gamma rays are diffracted by nearly perfect crystals of Si or Ge are measured using the GAMS4 facility. The lattice spacings of the diffracting crystals are determined elsewhere by measuring a crystal lattice spacing in terms of an optical wavelength and by measuring the small lattice spacing differences between crystal samples.

### 3.1 Principle of the Bragg Angle Measurements

**Fig. 1:**
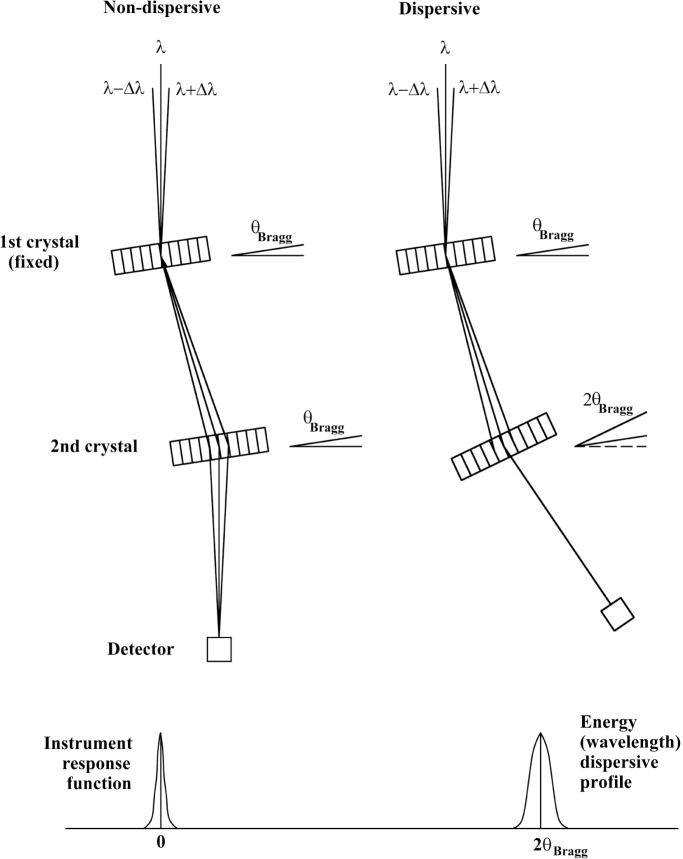
Schematic drawing of Bragg angle measurements using a two crystal spectrometer. The instrument response function is recorded using the non-dispersive geometry on the left and the gamma ray/instrument convolution function is recorded using the dispersive function on the right.

Figure 1 shows the crystal arrangements for the Bragg angle measurements and introduces the important concept of non-dispersive and dispersive geometries. Radiation from the source strikes the first crystal and all wavelengths that satisfy the Bragg condition are diffracted. The angular spread of the incoming beam is on the order of 1.3×10^−4^ rad for the GAMS4 facility. In the nondispersive geometry, the planes of the two crystals are parallel so that the second crystal simultaneously diffracts all wavelengths that are diffracted by the first crystal. By rocking the second crystal around the Bragg angle, a profile that is an accurate representation of the instrument response function is recorded. For a true nondispersive position (same lattice spacing and orders for both crystals) the recorded profile is insensitive to the spread in wavelength of the incoming radiation. The spectrometer is then configured to the dispersive geometry by keeping the first crystal fixed and rotating the second crystal through 2*θ*_Bragg_. In this geometry the second crystal does not simultaneously diffract all wavelengths diffracted by the first crystal. As the second crystal is rocked about the Bragg angle, the Bragg condition is satisfied sequentially for the wavelengths diffracted by the first crystal. Thus the recorded profile is a convolution of the instrument response function and the spread in wavelength of the incoming radiation. For many gamma rays, the spread in wavelength of the incoming radiation is very small so that the nondispersive and dispersive profiles are nearly identical. Accurate measurement of the angular distance between the nondispersive and dispersive geometries determines the Bragg angle.

### 3.2 GAMS4 Facility Layout

**Fig. 2:**
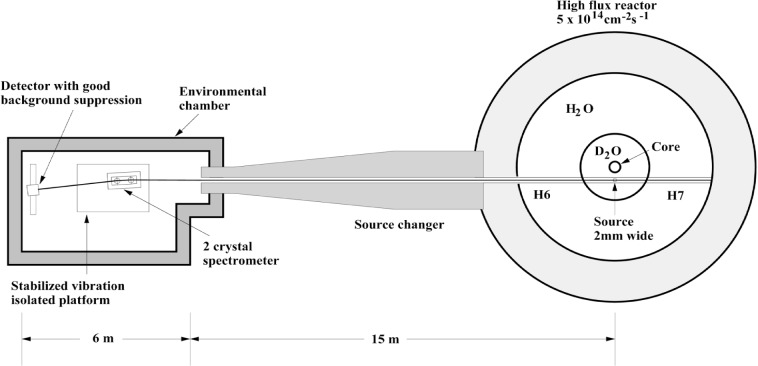
Layout of the GAMS4 precision gamma-ray spectroscopy facility on the ILL reactor floor.

The spectrometer is located on the ILL reactor floor so that it can accept radiation from sources placed in the “through tube” H6-H7 as shown in Fig. 2. The GAMS4 and GAMS5 spectrometers are located on the H6 and H7 sides of the through tube, respectively. The source changing mechanism and shield are located on the H6 side of the through tube between the reactor biological shield and the GAMS4 environmental chamber. Details concerning the through tube and the source changer are available in Ref. [15].

The GAMS4 environmental chamber is constructed out of concrete blocks lined with acoustical insulation. Inside the concrete blockhouse is a thermal enclosure that contains the vibration isolation platform, the spectrometer, and the collimators. Between the concrete blockhouse and the thermal enclosure is a heating system, which is used to stabilize the temperature of the spectrometer. Temperature variations of a few hundredths of a °C per day are typical. The detector is cryogenically cooled and is outside of the thermal enclosure. The vibration isolation platform is 2 m×3.5 m and permits moving the spectrometer and collimators out of the gamma-ray beam in order that the curved crystal spectrometer (GAMS23) which is located behind GAMS4 can be used. The position of the vibration isolation platform is stabilized with respect to the reactor floor using non-contacting proximity detectors and a electro-pneumatic servosystem similar to that described in Ref. [16]. All six degrees of freedom of the platform are stabilized to within a few microns and a few seconds for many months. During a measurement, the position of the spectrometer and the collimator on the vibration isolation platform are varied and the vibration isolation platform must respond to this change in loading.

### 3.3 Two Crystal Spectrometer

**Fig. 3:**
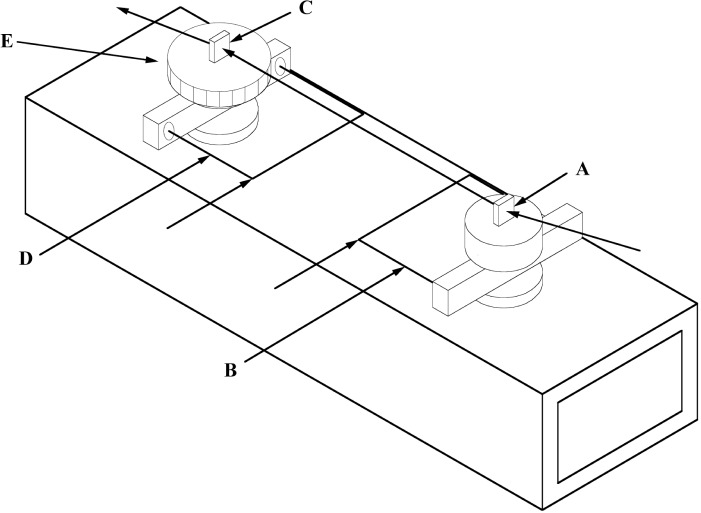
Schematic of the GAMS4 two crystal spectrometer. A: first crystal; B: first axis angle interferometer; C: second crystal; D: second axis angle interferometer; E: polygon for angle calibration.

The heart of the GAMS4 facility is the two crystal spectrometer that is shown in a very simplified way in Fig. 3. As was pointed out in Fig. 1, the Bragg angle is related to the relative angle between the two crystals. Therefore, rigid coupling of the two axes and the two interferometers that measure the rotations of the two axes is important. The spectrometer chassis is a cast iron hollow block 91 cm×30 cm×20 cm with 2.5 cm thick walls to which a precision optical square was permanently attached at the outset. All subsequent mechanical, optical, and crystal alignments are referenced to this five-sided optical square. The axes are defined by high precision bearings and are aligned with respect to the optical square and to each other within 1 s. The separation between the axes is approximately 53 cm.

The rotation of each axis is measured with a polarization encoded Michelson interferometer that has an angular sensitivity of a few × 10^−4^ s. The stationary optical elements are located on the spectrometer chassis between the two axes. Two corner cubes are mounted on an arm that is rigidly attached to the rotating crystal table and retro-reflect the light in each arm of the interferometer. A detailed description of the angle interferometers is given in Refs. [17, 18].

In order to convert interferometer fringes into angles, the interferometers are calibrated using an optical polygon and a photoelectric autocollimator to sense the directions normal to the faces of the optical polygon. Although the first axis is occasionally calibrated, the Bragg angle measurements as described above depend only on the calibration of the second axis. A 24-sided optical polygon (external angles ≈15°) is permanently mounted on this axis using matching face gears with 360 teeth so that the polygon can be indexed in multiples of 1° with respect to the axis and the corner cube arm. After measuring one of the external polygon angles in terms of interferometer fringes, the polygon is automatically indexed so that the next pair of faces can be viewed by the autocollimator and the corresponding external angle measured in fringes. After measuring all 24 external angles, the sum is constrained to equal 360°. The relative uncertainty of the angle calibrations is *u*_r_ ≈ 1×10^−7^. More details concerning the angle calibration are available in Refs. [17, 18].

### 3.4 GAMS4 Crystals

**Fig. 4:**
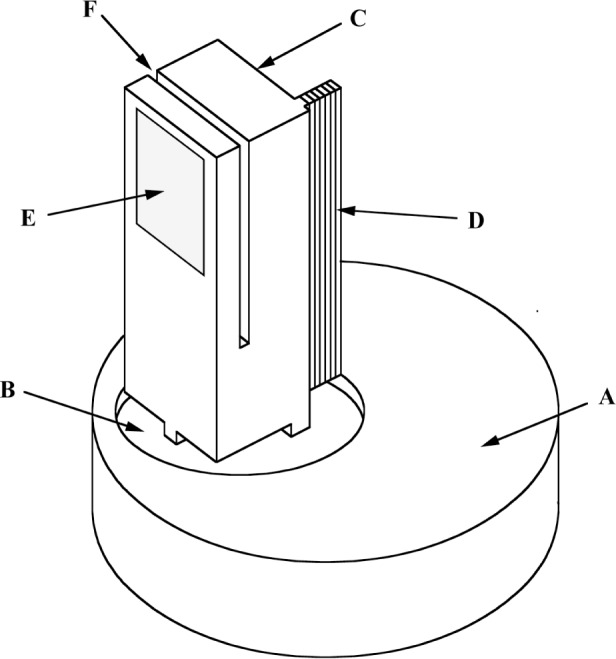
GAMS4 diffraction crystal. A: steel base; B: crystal base; C: diffraction crystal; D: gamma-ray diffracting planes; E: alignment mirror; F: strain relief cut.

Nearly perfect Si or Ge crystals diffract the gamma rays. Fig. 4 shows the crystal shape and mounting that has been used to obtain strain free specimens. The steel base provides a convenient way to attach the crystal to the spectrometer axis. The crystal base is the same material as the diffracting crystal and provides an essential material transition between the steel base and the diffracting crystal. The portion of the crystal that is used for diffraction is approximately 4 mm×50 mm which is slightly larger than the area of the sources that can be inserted in the reactor. The alignment mirror is a polished surface that serves as a reference to align the crystal planes parallel to the axis. The strain relief cut isolates the polished alignment surface that may be strained from the diffracting crystal planes. The three parts (steel base, crystal base, and diffracting crystal) are attached using low-shrink epoxy.

The lattice spacings of the GAMS4 crystals are measured with a relative uncertainty of *u*_r_ ≈ 5×10^−8^ using absolute and relative lattice parameter measurements. At the present time, four National Standards Laboratories contribute to these measurements. The Physikalisch-Technische Bundesanstalt (PTB), the Istituto di Metrologia “G. Colonnetti” (IMGC), and National Research Laboratory of Metrology (NRLM) are engaged in absolute lattice parameter measurements and PTB and the National Institute of Standards and Technology (NIST) are engaged in relative lattice parameter measurements. In order to clearly describe the lattice parameter measurements we present in Table 3 an example lattice parameter measurement using the actual values obtained for the 2.5 mm thick Si crystals. In the absolute lattice parameter measurements, the lattice spacing of a particular silicon crystal is measured in terms of an optical wavelength that provides a *d* for that particular crystal in meters. The results of the three national laboratories that have carried out these measurements are given in the second column of Table 3. Details of these very careful measurements are available in Refs. [19–21]. We have been provided with Si samples from the actual crystals used in the absolute lattice parameter measurements by two of the laboratories (IMGC and NRLM) and with a Si sample whose lattice parameter is known relative to the sample used for the absolute lattice parameter measurement by the third laboratory (PTB). Using the NIST lattice comparison spectrometer, relative lattice parameter measurements have been made between these three crystal samples and samples from the material used to make the gamma-ray crystals. Ref. [17] describes the NIST lattice comparison spectrometer in detail. In Table 3, column 3, the relative lattice parameter measurements for the GAMS4 2.5 mm thick crystals are given. The absolute lattice parameter measurements for the GAMS4 2.5 mm thick crystals are given in column 4 with the final average given in the last line. The uncertainty has been expanded to 5×10^−8^ to account for inconsistencies in the absolute and relative lattice parameter measurements. In general, the relative uncertainty of the crystal lattice spacing measurements is more than a factor of two less than the uncertainty of the Bragg angle measurements. Thus, significant improvement in the wavelength measurements is directly related to improvement in the Bragg angle measurements.

## 4. Wavelength Measurements

We now turn to a description of two recent GAMS4 experiments that were devoted to gamma-ray wavelength measurements that impact the fundamental constants. In the first experiment, a new value for the neutron mass was determined by directly measuring the deuteron binding energy. In the second experiment, the binding energy of ^36^Cl was determined by summing three transitions in the ^36^Cl cascade. As described in Sec. 2.1, this measurement can lead to a new value of the molar Planck constant, *N*_A_*h*.

### 4.1 Deuteron Binding Energy

The deuteron binding energy, *S*(d), was determined by measuring the 2.2 MeV gamma ray emitted in the reaction n + p ⇒ d + *γ* and correcting the measured energy for recoil. The 2.2 MeV transition connects the capture state and the ground state. The neutron mass, *m*(n), is obtained by expressing this reaction in atomic mass units, *m*(n) = *m*(^2^H) − *m*(^1^H) + *S*(d), and combining the deuteron binding energy with precision atomic mass measurements. Since a detailed description of this measurement is available in Ref. [22], the discussion presented here will be rather brief.

The gamma-ray source consisted of three thin walled graphite holders filled with ≈ 6 g of Kapton plastic (chemical formula N_2_H_10_O_5_C_22_). Kapton is a convenient source of hydrogen that is compatible with the reactor environment. The estimated total activity of the 2.2 MeV line at the beginning of the measurement was 1.6×10^13^ Bq. The gamma rays were diffracted by nearly perfect Si crystals having a thickness of 2.5 mm and the (220) crystal planes available for diffraction. This particular set of crystals was used in the example given in Sec. 3.4 above concerning the measurement of the crystal lattice spacing. Thus the lattice spacing values given in Table 3 are for this particular set of crystals. Gamma-ray profiles were recorded in three different configurations of the two axis spectrometer: (*m, n*) = (1,−2) and (1,2); (2, −1) and (2,2); (1, −3) and (1,3) where (*m,n*) denotes the reflection orders of the first (A) and second (B) crystals, respectively. The Bragg angles, peak count rates, and background count rates for the various crystal orders are given in Table 4. Note that no nondispersive profiles were recorded because the detector would be in the direct beam. The combination of small Bragg angles and low count rates is the reason precision measurement of this line is difficult. The profiles were fitted with a numerical profile generated using dynamical diffraction theory broadened with a Gaussian function to account for crystal imperfections, vibrations, and thermal motion of the atoms in the source. The adjustable parameters in the fit are the position, intensity, background, and Gaussian width. The sequence of profile recording was typically −cw, +cw, +ccw, −ccw where − and + refer to the less and more dispersive profiles, respectively, and cw and ccw indicate clockwise and counter clockwise rotation of the axis. Bragg angles were obtained from the profile fringe values using an angle calibration recorded immediately before or after the measurements. For each configuration, multiple (≥25) determinations of *θ*_Bragg_ were made.

Wavelengths were determined by combining the measured Bragg angle and the crystal lattice spacing using the Bragg condition for diffraction, *λ* = 2*d*sin*θ*. The wavelength of the 2.2 MeV gamma ray was determined to be *λ*_meas_ = 5.576 712 99(99)×10^−13^ m. This measured gamma-ray wavelength must be corrected for recoil to obtain a wavelength, λ*, that corresponds to the binding energy of the deuteron, *S*(d). The value obtained for λ* is λ* = 5.73 409 78(99)×10^−13^. The deuteron binding energy, *S*(d), can be expressed in atomic mass units and eV by using the inverse meter to atomic mass unit [(Eq. (5)] and the inverse meter to eV conversion factors. The results are: *S*(d) = 2.388170 07(42)×10^−3^u and *S*(d)=2 224 566.14(41)eV. Finally the mass of the neutron is obtained from the equation *m*(n) = *m*(^2^H)−*m*(^1^H) + *S*(d) where the most precise value for *m*(^2^H)−*m*(^1^H) is available from Ref. [2], *m*(^2^H)−*m*(^1^H) = 1.006 276 746 30(71) u. The result is *m*(n) = 1.008 664 916 37(82) u.

### 4.2 ^36^Cl Binding Energy

Building on the experience gained during the 2.2 MeV gamma-ray measurement, we turned our attention to the measurement of the gamma rays produced in the reaction n + ^35^Cl ⇒ ^36^Cl + *γ*’s (8.6 MeV). The ^36^Cl binding energy, *S*(^36^Cl), is most conveniently obtained by summing three gamma rays, one of which has an energy near 6 MeV. The high capture cross section of ^35^Cl (*σ*= 43.3×10^−24^ cm^2^) allows it to offer the most intense gamma cascade among those light nuclei with level structures suited to measurement of the neutron separation energies. The complementarity of the high energy gamma-ray and atomic mass measurements can be seen by expressing this reaction in atomic mass units, *m*(n)−*S*(^36^Cl) = *m*(^36^Cl)−*m*(^35^Cl). The ^36^Cl binding energy and the neutron mass determined above provide a measure of the atomic mass difference *m*(^36^Cl)−*m*(^35^Cl). Conversely, if the atomic mass difference *m*(^36^Cl)−*m*(^35^Cl) is known, then a value for the molar Planck constant, *N*_A_*h*, can be extracted as explained in Sec. 2.1 above.

**Fig. 5:**
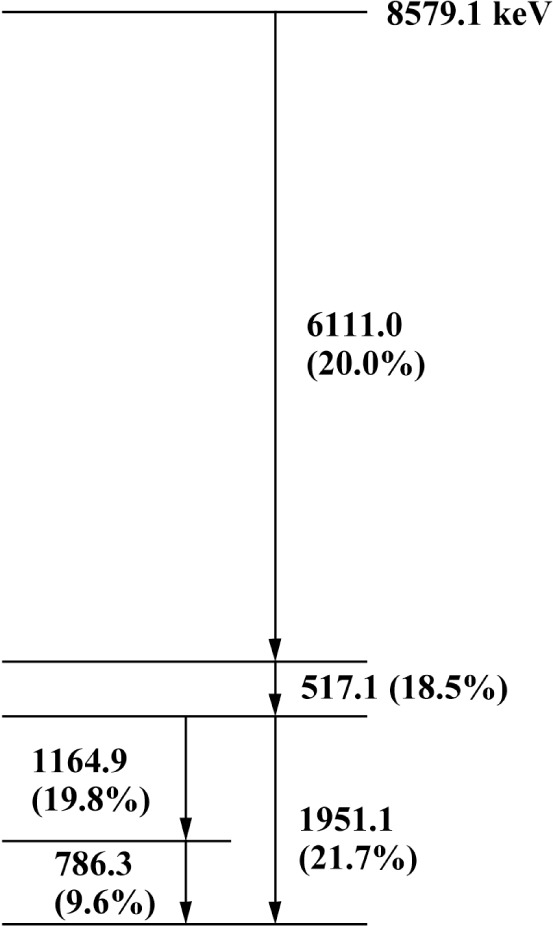
Decay scheme of ^36^Cl showing only the transitions measured in this work. The numbers in parentheses are the number of *γ* rays per 100 neutron radiative captures.

**Fig. 6:**
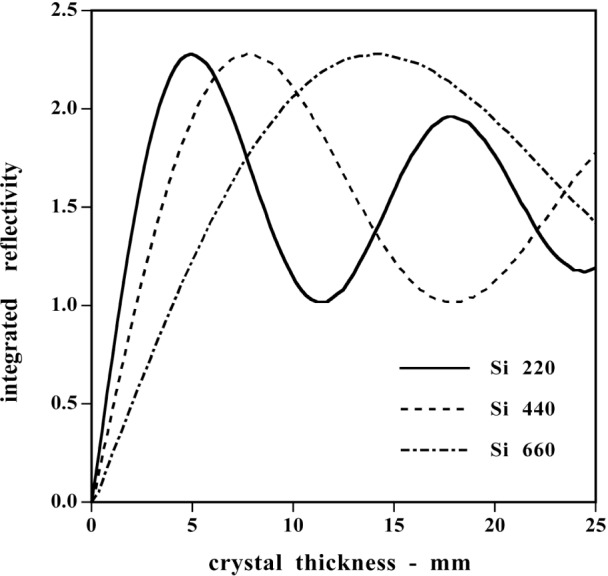
Theoretical integrated reflectivity at *E* = 6.1 MeV for three low-order single crystal Si reflections.

The gamma-ray lines that were measured to determine the ^36^Cl binding energy are shown in the energy level diagram in Fig. 5. The binding energy is obtained as the sum of the 517.1, 1951.1, and 6111.0 keV lines after correction for recoil. The 786.3 and 1164.9 keV lines measure the same interval as the 1951.1 keV line and provide a test of the internal consistency of our measurements. Our earlier measurements of the lines below 2 MeV [23] were made with the original installation of GAMS4. Significant improvements made during the intervening 13 years permit realization of higher accuracy in the new results reported here. The chlorine gamma radiation was produced by placing three graphite target holders containing natural high purity NaCl near the reactor core in a neutron flux of ≈ 5×10^14^ cm^−2^ s^−1^. Each target contained 1.5 g of NaCl and had a volume of 2 mm×35 mm×25 mm. The estimated total activity of the ^36^Cl was about 6×10^14^ Bq. Two sets of crystals were used for these measurements. The first set, the same crystals used for the deuteron binding energy measurement, had two crystals of equal thickness (2.5 mm). The second set had two crystals of unequal thickness (4.41 mm and 6.95 mm). The thinner crystals are more appropriate for the lower energy lines, while the thicker crystals are superior for the 6.1 MeV line. This fact is illustrated in Fig. 6, which shows the integrated reflectivity vs crystal thickness for three orders at 6.1 MeV. For low count rate measurements, the intensity can be significantly increased by carefully choosing the crystal thickness. The lattice spacing of the 4.4/6.9 mm crystal set was determined using a procedure that was similar to that employed for the 2.5 mm set. The relative uncertainty of the measured lattice spacing of the 4.4/6.9 mm crystal set is *u*_r_ ≈ 5×10^−8^.

Each line was measured in at least two orders that were chosen on the basis of high crystal reflectivity and small diffraction width. The energies, crystals, orders, and approximate Bragg angles are given in Table 5. The small Bragg angle (0.061° in first order) through which the 6.1 MeV radiation is diffracted places very stringent requirements on the accuracy of the angle measurements in order to obtain binding energy measurements with a relative uncertainty *u*_r_ < 1×10^−6^

**Fig. 7:**
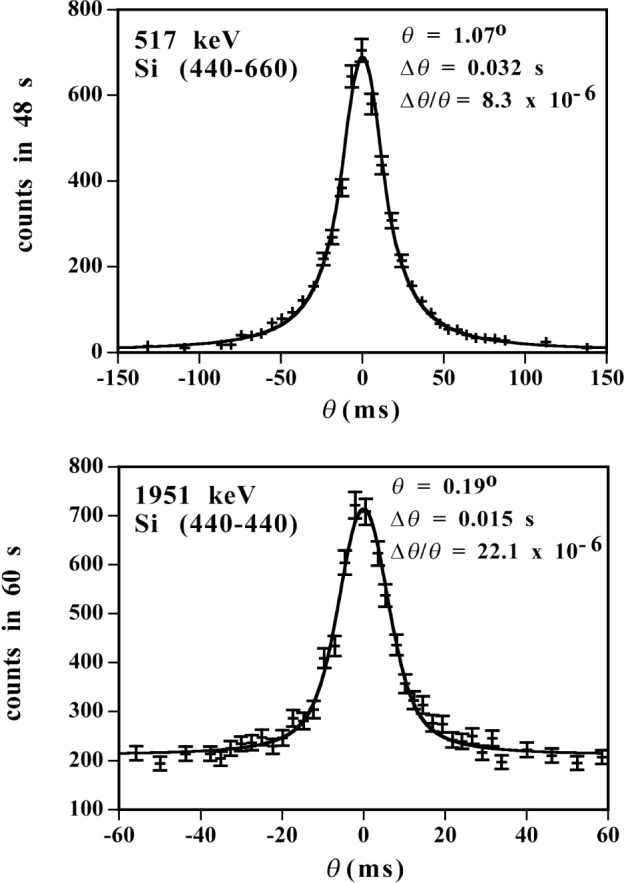
Typical profiles and fits for the 517 keV and 1951 keV lines in ^36^Cl.

**Fig. 8:**
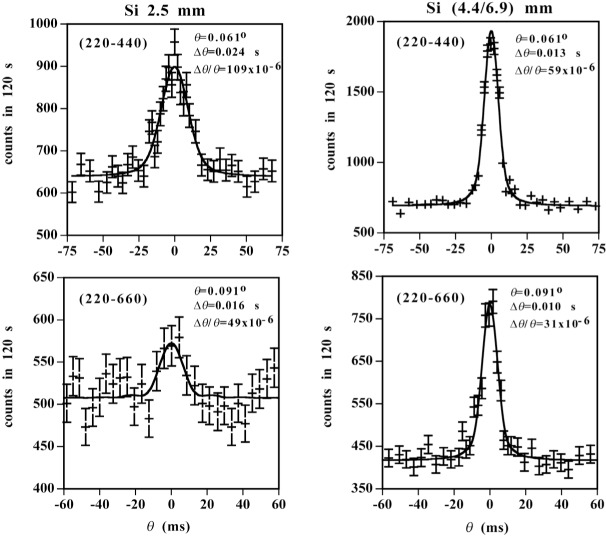
Typical profiles and fits for the 6.1 MeV transition in ^36^Cl. Note that the profiles on the left and right were recorded with the 2.5 mm and 4.4/6.9 mm thick crystals, respectively.

The profile analysis and the extraction of Bragg angles are almost identical to that described above for the 2.2 MeV gamma ray. The only difference is that the small Gaussian broadening of the lower energy lines includes a contribution due to recoil following the emission of a primary gamma ray in addition to the contributions mentioned above. Typical profile scans and the fit for a representative number of energies and orders are given in Figs. 7 and 8. In Fig. 8, it is important to note that the profiles obtained with the thicker crystals are not only more intense, but also significantly narrower. For each order and transition, multiple (>5) determinations of *θ*_Bragg_ were made.

Wavelengths are determined by combining the measured Bragg angles and the lattice spacings using the Bragg condition for diffraction, *λ* = 2*d*sin*θ*. Before publishing final wavelength values for the five Cl lines and the ^36^Cl binding energy, further data analysis of the Bragg angle measurements and additional lattice spacing measurements on the 4.4/6.9 mm crystals must be completed. At the present state of the data analysis, it appears that the relative uncertainty of the lower energy lines is ≈ 2 to 3×10^−7^, of the 6.1 MeV line is 4×10^−7^, and of the ^36^Cl binding energy is 3×10^−7^. These uncertainties along with the equation for the reaction in atomic mass units, *m*(n)−S(^36^Cl) = *m*(^36^Cl)−*m*(^35^Cl), allows us to estimate the uncertainty of the two sides of this equation. The relative uncertainty of the left-hand side is {[*m*(n) = 1.008 664 916 37(82) u]−[*S*(^36^Cl) = 0.009 211 000(3) u (approximate value with a reliable uncertainty estimate)]} ≈ 3×10^−9^. The relative uncertainty of the right hand side is {[m(^36^Cl) = 35.968 306 945(83)]−[*m*(^35^Cl) = 34.968 852 707(42)]} ≈ 9×10^−8^ [24]. In this case, the gamma-ray measurements lead to a more accurate value for the m(^36^Cl)−*m*(^35^Cl) mass difference than is currently available from the atomic mass measurements. When more precise atomic mass measurements of Cl become available, then this same equation can be used to determine a value for the molar Planck constant, *N*_A_*h.*

## Figures and Tables

**Table 1:** Major approaches to the determination of the fine structure constant, *α*. For definitions of the constants appearing in these equations, refer to the references listed with each equation

$a_{e}=C_{1}\left(\frac{\alpha }{\pi }\right)+C_{2}{\left(\frac{\alpha }{\pi }\right)}^{2}+C_{3}{\left(\frac{\alpha }{\pi }\right)}^{3}+\ldots$	(11)	*a*_e_ = *g*_e_−1 anomalous moment [5]	*u*_r_ ≈ 4×10^−9^
$\alpha^{-1}=\left[\frac{2}{\mu_{0}c}{\left\{R_{K}\right\}}_{\text{LAB}}\left(\frac{\Omega_{\text{LAB}}}{\Omega }\right)\Omega \right]$	(12)	von Klitzing constant *R*_K_ = *h*/*e*^2^ [9]	*u*_r_ ≈ 3×10^−8^
$\alpha^{-1}={\left[\frac{\mu_{p}^{\prime }}{\mu_{B}}{\left\{\frac{2e}{h}\right\}}_{\text{LAB}}\frac{{\left\{R_{K}\right\}}_{\text{LAB}}}{2\mu_{0}R_{\infty }{\left\{\gamma_{p}^{\prime }\right\}}_{\text{LAB}}}\frac{\mathrm{kg}}{s^{2}A^{2}}\right]}^{1/3}$	(13)	von Klitzing, Josephson junction, and $\gamma_{p}^{\prime }$ [10,11]	*u*_r_ ≈ 6×10^−8^
$\alpha^{-1}={\left[\left(\frac{2R_{\infty }}{c}\right)\left(\frac{A_{r}(n)}{A_{r}(e)}\right)\left(\frac{h}{m_{n}}\right)\right]}^{1/2}$	(14)	*h/m*_n_ = *λv* Krüger et al. [12]	*u_r_* ≈ 4×10^−8^
$\alpha^{-1}={\left[\left(\frac{2R_{\infty }}{c}\right)\left(\frac{A_{r}(\mathrm{Cs})}{A_{r}(e)}\right)\left(\frac{h}{m_{\mathrm{Cs}}}\right)\right]}^{1/2}$	(15)	*h/m*_Cs_ *= λv* Chu et al. [13]	*u*_r_ ≈ 4×10^−8^ (preliminary)

**Table 2:** Uncertainty contributions to a determination of the molar Planck constant, *N*_A_*h*, and the fine structure constant, *α*, for several candidate nuclei. For each case the relative uncertainties of the mass doublet ratio, *A*_r_(*^A^*XD^+^)/*A*_r_(*^A^*^+1^XH^+^), and $\lambda_{A+1}^{*}$ are assumed tobe *u*_r_ = 5×10^−11^ and *u*_r_ = 2×10^−7^, respectively

	*S* (*^A^*^+1^X)	*u*_r_−*N*_A_*h*	*u*_r_−*N*_A_*h*	*u*_r_−*N*_A_*h*	*u*_r_−*α*	
*^A^*+1X	(MeV)	GAMS4	Δ*M*	total	total	Notes
^15^N	10.8	2.6×10^−7^	8.7×10^−8^	2.7×10^−7^	1.4×10^−7^	This presents an intensity problem for GAMS4
^29^Si	8.5	2.8×10^−7^	2.2×10^−7^	3.6×10^−7^	1.8×10^−7^	Not yet measured with GAMS4
^33^S	8.6	2.8×10^−7^	2.5×10^−7^	3.7×10^−7^	1.9×10^−7^	Has been measured with GAMS4
^36^Cl	8.6	2.8×10^−7^	2.7×10^−7^	3.9×10^−7^	1.9×10^−7^	Has been measured with GAMS4 (see Sec. 4.2); ^36^Cl is slightly Radioactive which complicates the trap experiment
^49^Ti	8.1	2.8×10^−7^	3.9×10^−7^	4.9×10^−7^	2.4×10^−7^	Not yet measured with GAMS4

**Table 3:** Lattice spacing of the ILL2.5 crystals

	*d*(220)^a^	$\frac{\text{ILL}2.5-\text{column}2}{\text{ILL}2.5}$	*d*(220)^a^
	Absolute lattice crystal (pm)		ILL2.5 (pm)
PTB	192.0 155 63(12)	1.7(1.7) 10^−8^^b^	192.0 155 66(12)
IMGC	192.0 155 51(5)	8.6(1.0) 10^−8^^c^	192.0 155 676(54)
NRLM	192.0 155 87(10)	3.4(1.0) 10^−8^^c^	192.0 155 93(10)

Mean *d*(220) ILL2.5			192.0 155 723(96)

a *t* = 22.5 °C in vacuum.

b Includes PTB and NIST comparisons.

c Direct NIST comparisons

**Table 4:** Crystal orders, Bragg angles, and count rates for the *d* binding energy measurement

A crystal		B crystal		Peak count rate (s^−1^)	Background count rate (s^−1^)
Order (*m*)	Bragg angle (deg)	Order (*n*)	Bragg angle (deg)		
1	0.083	−2	0.166	1.02	0.035
1	0.083	2	0.166	0.78	0.078
1	0.083	−3	0.249	0.21	0.008
1	0.083	3	0.249	0.18	0.079
2	0.166	−1	0.083	0.90	0.048
2	0.166	2	0.166	0.29	0.063

**Table 5:** Energies, crystals, orders, and Bragg angles for the ^36^Cl binding energy measurement

		A crystal		B crystal	
Energy (keV)	Thickness (mm)	Order (*m*)	Bragg angle (deg)	Order (n)	Bragg angle (deg)
517.1	2.5	2	0.716	±2	0.716
517.1	2.5	2	0.716	±3	1.074
786.3	2.5	1	0.235	±1	0.235
786.3	2.5	3	0.706	±3	0.706
1164.9	2.5	2	0.318	±2	0.318
1164.9	2.5	3	0.477	±3	0.477
1951.1	2.5	1	0.085	±2	0.190
1951.1	2.5	2	0.190	±2	0.190
6111.0	2.5; 4.4/6.9	1	0.030	+1	0.030
6111.0	2.5; 4.4/6.9	1	0.030	+2	0.061
6111.0	2.5; 4.4/6.9	1	0.030	±3	0.091
